# Erasing stigmas through storytelling: why interactive storytelling environments could reduce health-related stigmas

**DOI:** 10.1080/24735132.2024.2306771

**Published:** 2024-02-05

**Authors:** Niko Vegt, Valentijn Visch, Wilbert Spooren, Elisabeth F. C. van Rossum, Andrea W. M. Evers, Annemiek van Boeijen

**Affiliations:** a Faculty of Industrial Design Engineering, Delft University of Technology, Delft, The Netherlands; b Faculty of Arts, Radboud University, Nijmegen, The Netherlands; c Obesity Center CGG, Erasmus MC, University Medical Center Rotterdam, Rotterdam, The Netherlands; d Department of Internal Medicine, division of Endocrinology, Erasmus MC, University Medical Center Rotterdam, Rotterdam, The Netherlands; e Faculty of Social and Behavioral Science Leiden University, Leiden, The Netherlands; f Faculty of ESHPM, Erasmus University, Rotterdam, the Netherlands

**Keywords:** Health-related stigmas, storytelling, weight stigma, design

## Abstract

In this article we describe how designers can apply storytelling to reduce health-related stigmas. Stigma is a pervasive problem for people with illnesses, such as obesity, and it can persistently hinder coping, treatment, recovery, and prevention. Reducing health-related stigma is complex because it is multi-layered and self-perpetuating, leading to intertwined vicious circles. Interactive storytelling environments can break these vicious circles by delimiting the narrative freedom of stigma actors. We theoretically explain the potential of interactive storytelling environments to reduce stigma through the following seven functions: 1) expose participants to other perspectives, 2) provide a protective frame, 3) intervene in daily conversations, 4) persuade all stigma actors, 5) exchange alternative understandings, 6) elicit understanding and support for stigma victims, and 7) support stigma victims to cope with stigmatization. We elaborate on these functions through a demonstration of an interactive storytelling environment against weight stigma. In conclusion, this article is a call on designers for health and wellbeing, scientists, and practitioners from various disciplines to be sensitive to the pervasiveness of stigma and to collaboratively create destigmatizing storytelling environments.

## Introduction

Stigma is a pervasive problem for people with illnesses (Weiss, Ramakrishna, and Somma 2006), such as HIV/AIDS, skin diseases, depression, and obesity. Victims of health-related stigmatization give testimonies of feeling and being bullied, secluded, and violated. As a result, they continuously feel pressure to act normal, make a good impression, and provide explanations for supposed shortcomings (Goffman 1963). This places a huge burden on people and it can lead to negative physical and psychological health outcomes (Pachankis 2007). At the same time, perpetrators of stigmatization are often not fully aware of their stigmatizing behaviour and its negative effects. They ‘just want the best for someone’, make assumptions about someone’s condition, and are sometimes just ill-informed about a disease.

Health-related stigmatization is a complex phenomenon involving many actors, motivations, and effects that negatively impact the health of stigma victims (Quinn and Chaudoir 2009; Van Beugen et al. 2017). Health-related stigma not only manifests itself in interpersonal interaction. It also manifests itself internally (intrapersonal) and structurally. Stigmatized individuals themselves often internalize parts of the biases and stereotypes leading to self-stigma (Richman and Lattanner 2014). Structural health-related stigma can be found in health insurance policies that exclude certain illnesses, reduced chances on the labour market, and health inequalities among populations (Hatzenbuehler 2016). Hence, health-related stigma can persistently manifest itself in nearly all facets of people’s lives and this negatively influences the health of stigma victims and can lead to health inequalities (Pascoe and Smart Richman 2009; Paradies, Luiz Bastos, and Priest 2016). Some argue that stigma also serves a purpose as a motivator for healthy behaviour change (Bayer 2008), yet health-related stigma does more harm than good and should thus be reduced.

Unfortunately, reducing health-related stigma is a complicated endeavour. Stigmas are generally very persistent because motivations to stigmatize are often deeply ingrained in people’s norms and beliefs, and in societal structures (Scambler 2009). The multifaceted and multi-layered nature of stigmatization (e.g. labelling, distancing, discriminating on an intrapersonal, interpersonal, structural level) makes a centralized top-down approach, such as advertisement campaigns, ineffective in the long term (Link and Phelan 2001). Additionally, stigma is often subject to psychological mechanisms that perpetuate stigmatizing attitudes and behaviours (Brewis 2014).

We propose storytelling to be a driving force against stigmatization because, on a structural level, health-related stigmas can be characterized as a cultural web of narratives (Meretoja 2017) and master narratives have been widely used to uncover societal oppressive structures against marginalized communities (Syed 2015). Moreover, on the intrapersonal level, narratives are strongly related to personal identity formation by which individuals position themselves in conversations (Bamberg 2011).

**Figure 1.**
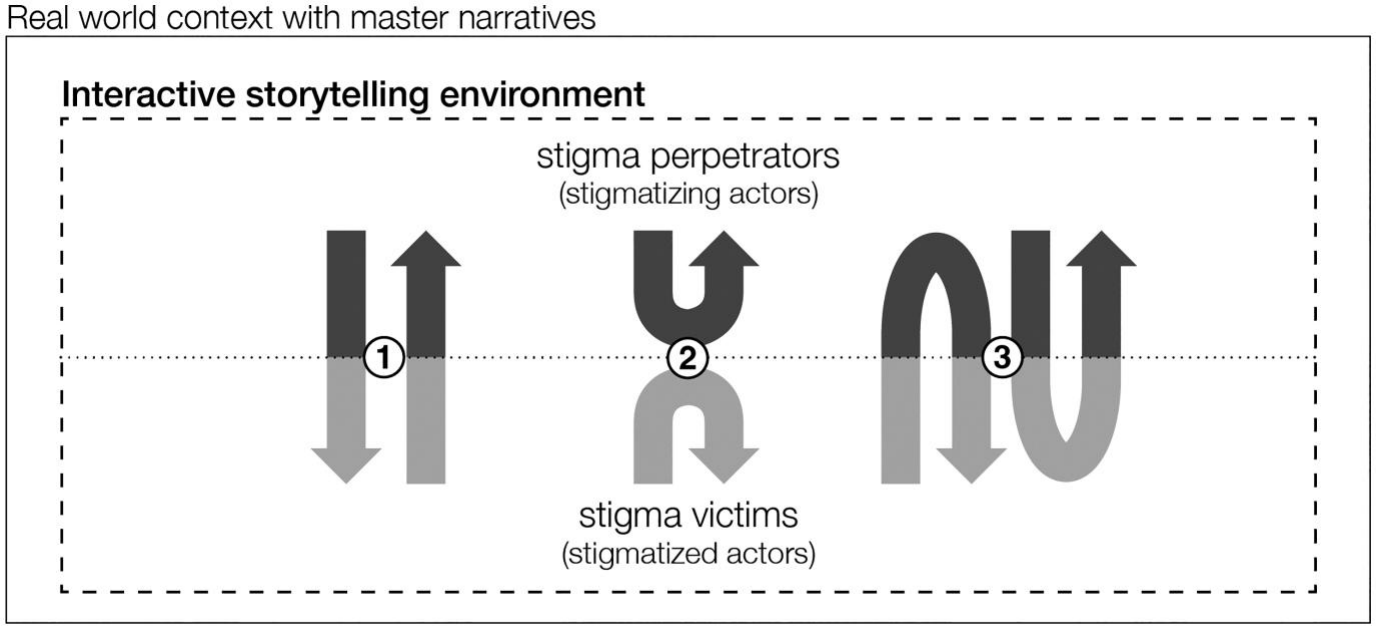
We propose to design interactive storytelling environment that shape the conceptual storytelling space of stigma actors to motivate them to 1) appropriate, 2) reflect on, and 3) co-create their stories.

Rather than intervening on single narratives, as in conventional campaigns against stigma, we suggest targeting the exchange of narratives, i.e. the story*telling*. Stigma actors may be invited to create and feel part of destigmatizing stories by safe and unprejudiced social environments with confined degrees of freedom in telling stories and interacting with them. As visualized in Figure 1, ‘interactive storytelling environments’ with a designed conceptual storytelling space, i.e. the set of all possible stories that participants can tell, may provoke stigma perpetrators and stigma victims to 1) appropriate their stories for the other group, 2) reflect on one’s own stories, and 3) adapt one’s stories in collaboration with the other group.

In this article, we explore the potential of designing interactive storytelling environments to reduce health-related stigmatization by identifying destigmatizing functions of story content and story interactions and applying them to an intervention. In the first half of the article, we review the literature on stigma, with weight stigma as an exemplary case, to better understand its pervasive and self-perpetuating nature. In the second half of the article, we define a research agenda about designing destigmatizing functions of storytelling and describe a serious game against weight stigma to demonstrate the destigmatizing potential of interactive storytelling environments.

## Stigma as a psychosocial phenomenon

Stigma is a visible or invisible mark of disapproval, allowing ‘insiders’ to identify and disassociate from ‘outsiders’ (Falk 2001). Goffman (1963) describes the day-to-day experiences of outsiders as ‘managing impressions’ and ‘managing information’. For example, people that are stigmatized by their body size particularly recall managing impressions, such as making sure to dress nicely and not eating in public (Lewis et al. 2011). Link and Phelan (2001) define stigma as a co-occurrence of labelling, stereotyping, separating, status loss, and discrimination in a power situation that allows these processes to unfold. The power difference between a stigmatized group and the group that stigmatizes is important to point out, because stigmas are persistent when they exist within groups that have the power to find new strategies to maintain the disadvantageous status of the stigmatized group. As long as motivations for stigmatization prevail in dominant groups, combatting mediating processes is ineffective (Hatzenbuehler, Phelan, and Link 2013).

Stigmatization should be reviewed on an intrapersonal, interpersonal, and structural level to fully understand it (Cook et al. 2014). Health-related stigma that manifests itself in interpersonal interaction is often easy to identify, such as bullying, negative commenting, or excluding (Hebl and Dovidio 2005). Yet for people with a chronic illness, intrapersonal or self-stigma is particularly salient (Durso and Latner 2008; Kranke et al. 2011). They often go through a process of understanding the causes of their illness and thus regularly fail in their attempts to recover or manage it. This can lead to reduced self-esteem and self-worth and in effect self-stigma in the form of inhibiting or isolating oneself (Corrigan, Larson, and Rüsch 2009).

Structural stigmatization can be found in socioeconomic conditions, cultural norms, and institutional policies (e.g. Hatzenbuehler and Link 2014). For example, people with obesity have on average lower incomes (Giskes et al. 2010) and weight loss services are often not reimbursed by the health insurance (Puhl and Heuer 2009). Stangl et al. (2019) explain that such structural discrimination on health conditions can be found in organizations, communities, and public spaces. Thus, in this article, ‘stigma actor’ refers to people that are the victim or perpetrator of stigmatization as well as non-human actors through which health-related stigmas are expressed, such as health insurance systems, cultural master narratives, and the media. Moreover, our use of ‘victim’ or ‘perpetrator’ in this article refers to the role of a stigma actor in a particular stigmatizing situation, rather than being or feeling a victim or perpetrator in general.

To get a more concrete understanding of the multiple levels and actors of health-related stigma, we dive deeper into the manifestations, causes, and effects of weight stigma. Weight stigma is a valuable case because many recent studies show direct negative health consequences of stigmatization, yet means to reduce weight stigma are scarce (Pearl 2018). Such a comprehensive understanding of health-related stigmas allows us to define mechanisms that could erase it.

### Weight stigma

People with obesity are stigmatized in many ways and settings. They are called ‘lazy, unmotivated, lacking in self-discipline, less competent, non-compliant, and sloppy’ (Puhl and Heuer 2009, 941) in employment, healthcare, education, interpersonal relationships, and media settings. As obesity is visible, these stereotypes easily lead to separation and discrimination in the form of gazing, others making decisions for you, negative comments, being ignored or rejected, and bullying or even physical violation (De Brún et al. 2014; Lewis et al. 2011). Next to labelling by others, many people with obesity have the same anti-fat attitudes as slender people (Crandall 1994). This self-stigma is expressed in the form of a negative body image (Harriger and Thompson 2012), feeling guilty or weak (Lillis et al. 2010), distancing from others that have obesity (Durso and Latner 2008), and reduced motivation in weight loss efforts (Corrigan, Larson, and Rüsch 2009; Hunger et al. 2015).

Losing weight is complex due to the wide variety of causes for weight gain. To some extent, it is caused by unhealthy nutrition and too little physical activity, yet a substantial part of the variation in body weight is determined by other determinants, such as genetic composition and reduced access to healthy food (Kolata 2007; Vandenbroeck, Goossens, and Clemens 2010). Moreover, there are many cultural and psychosocial factors that contribute to obesity, such as role models (Lau, Lee, and Ransdell 2007), body image preferences (Becker et al. 1999), preoccupation with dieting (Bacon and Aphramor 2011), stress (Tomiyama 2014), and depression (Milaneschi et al. 2019). Also somatic causes may play a role, e.g. hormonal causes, medication with weight gaining side-effects and many other factors (van der Valk et al. 2019).

On a societal level, people with obesity are generally personally blamed for their weight (Saguy and Gruys 2010). This is often based on the false assumption that personal factors such as a sedentary lifestyle, poor eating behaviours, and psychological problems are the only causes of obesity (Puhl et al. 2015). Moreover, it is often thought that losing weight is easy, whereas compelling evidence exists that mechanisms in the body and brain counteract weight loss when a person developed obesity (Pucci and Batterham 2020). This leads to structural discrimination in practices such as not fitting in seats or clothing (Brewis et al. 2017), negative media portrayals (Heuer, McClure, and Puhl 2011), lower job opportunities (Brewis 2014; Puhl and Heuer 2010), insurances not covering weight loss treatments, and absence of anti-stigma legislation (Pearl 2018).

Thus, living with obesity and dealing with the accompanying stigma can negatively interfere with nearly all facets of people’s lives, in particular, in cultural contexts where a slender body is encouraged in many forms, e.g. social media, fashion, education. This example of weight stigma highlights the pervasive nature of health-related stigma.

### Vicious circles of health-related stigma

We dive deeper into the reasons why health-related stigma is so pervasive to arrive at recommendations for sustainable stigma reduction. One explanation are the persistent motivations to stigmatize. Phelan, Link, and Dovidio (2008) describe three generic motivations to stigmatize: 1) exploitation, 2) enforcement of social norms and 3) avoidance of disease. The above-described manifestations of health-related stigma and weight stigma mainly provide evidence for norm enforcement and disease avoidance. Monaghan (2017) suggests that personally blaming people for obesity is rarely challenged due to a predominant ideology of personal responsibility, marketization and rolling back of the welfare state. This fits in a broader practice of stigmatizing people that burden the healthcare system and holding them personally accountable for this burden (Brelet et al. 2021).

Next to attributing chronic illnesses to personal responsibility, other motivations to stigmatize are cultural norms of physical attractiveness and pathogen avoidance. In many cultures there is a preference for thinness (Crandall 1994) and concerns of contagion may arise due to the widespread knowledge of health problems associated with certain chronic illnesses (Pearl 2018). Consequently, self-stigma may arise because the overall norms of being healthy may be stronger than self-interest. Moreover, people with a chronic illness often protect their self-image by distancing themselves from the negative label (De Brún et al. 2014), viewing their illness as a temporary condition (Durso and Latner 2008), and not identifying with others that are ill (Tomiyama et al. 2015). As a result, the stigma prevails because it is not challenged by anyone.

**Figure 2.**
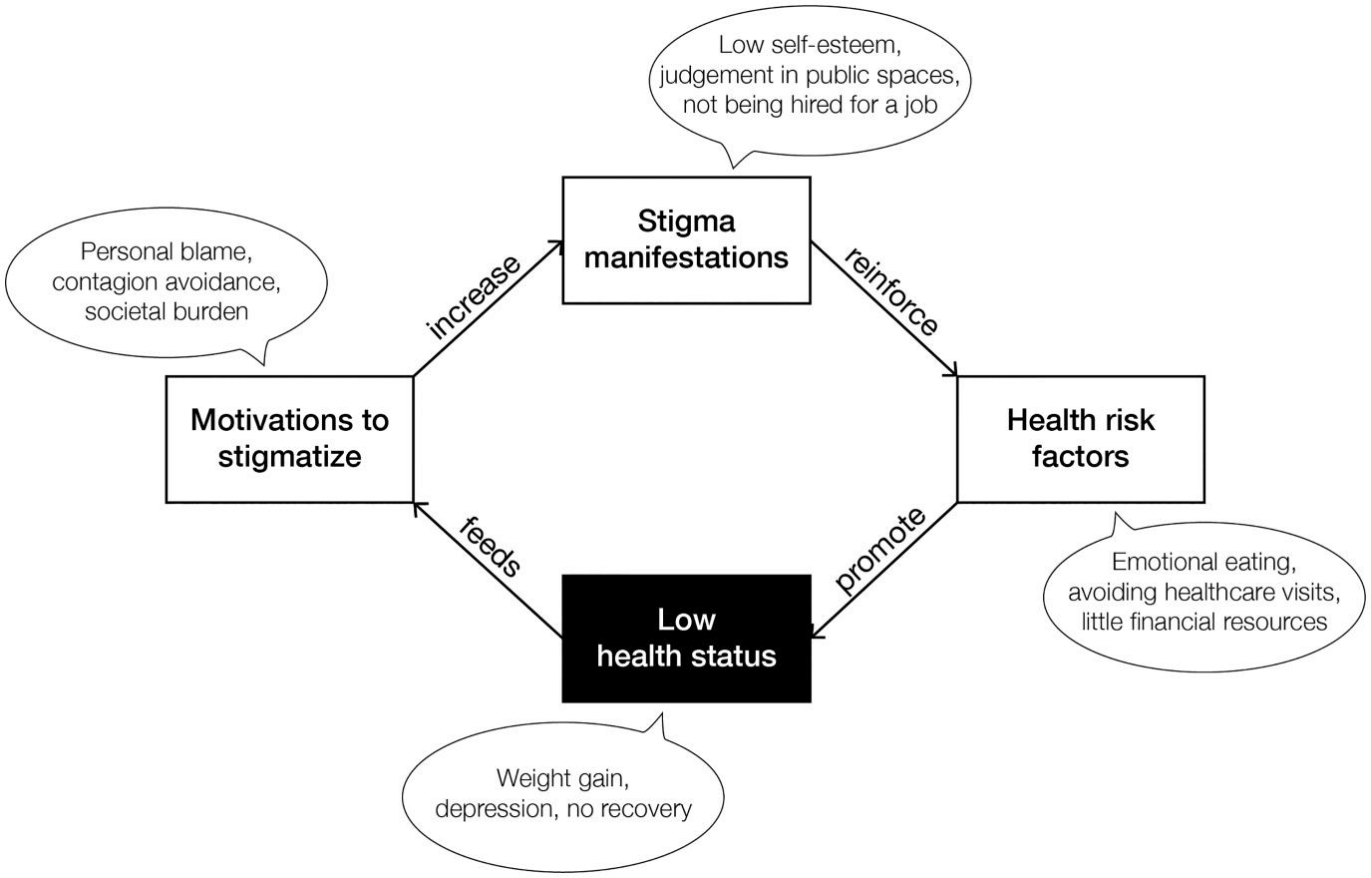
The health-related stigma perpetuation model – a vicious circle (with obesity-related examples).

Health-related stigma is also pervasive because it can cause health disparities and vice versa (Hatzenbuehler, Phelan, and Link 2013), leading to a self-perpetuating cycle (see Figure 2). For example, stigmatization seems one of the causes for weight gain and a barrier for weight loss due to its negative social and psychological effects (Papadopoulos and Brennan 2015; Puhl and Suh 2015). If a person with obesity encounters stigma, they may start to feel more alone and isolated. This may cause depression and stress, and depression and stress cause negative health outcomes, such as weight gain (Jackson and Steptoe 2018; Tomiyama 2014). As a result, stigmatization can lead to a negative loop in which causes and effects of weight gain get intertwined.

**Figure 3.**
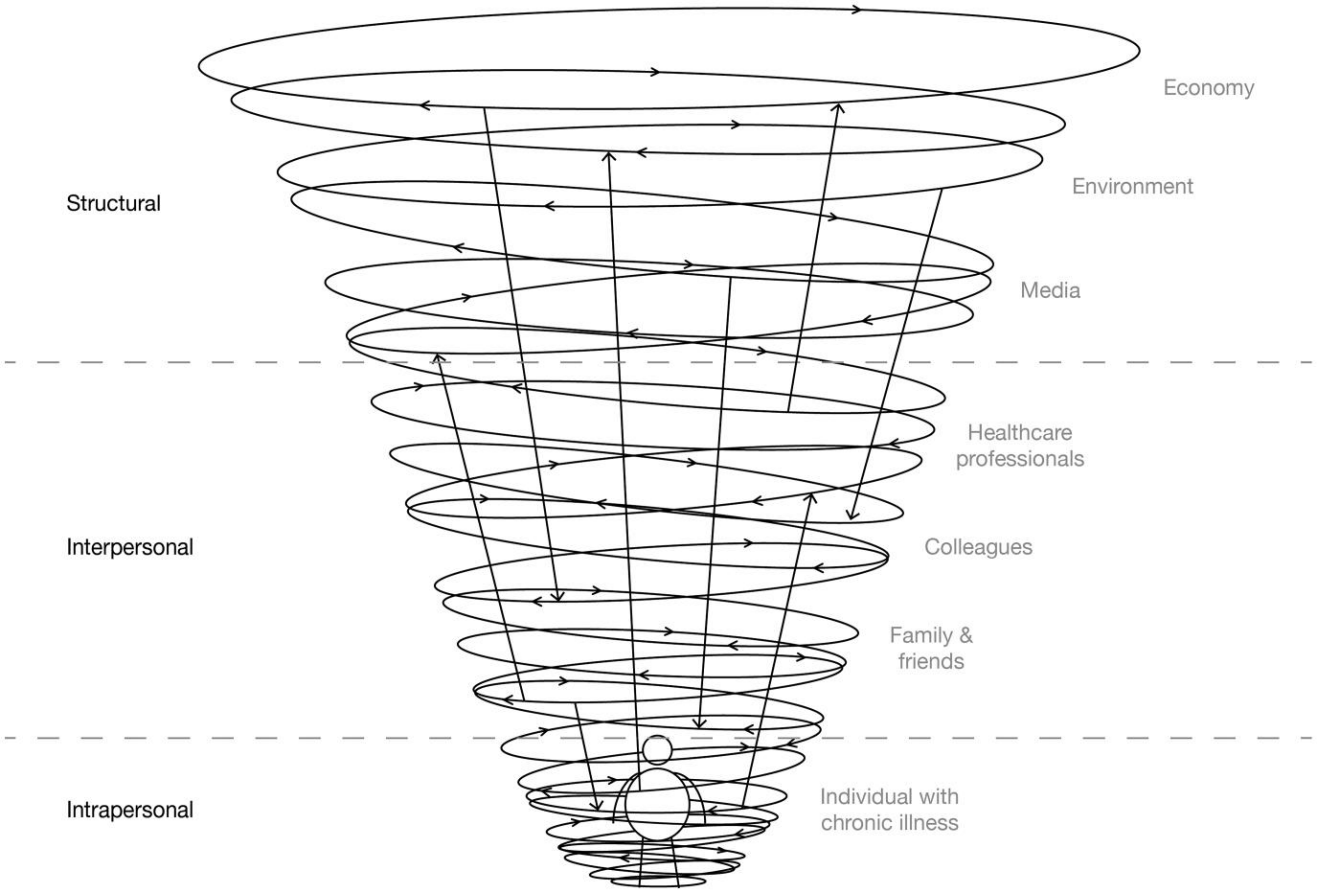
People with chronic illnesses can experience interrelated vicious circles of health-related stigma on three levels. Examples of human and non-human actors are visible on the right.

A vicious circle of health-related stigma starts when the effects of stigmatization negatively influence the victim’s physical or psychological health, and when the worsened health condition feeds motivations to stigmatize. Such vicious circles can be identified in intrapersonal, interpersonal, and structural stigmatization and are interlinked, as visualized in Figure 3. For example, on an intrapersonal level, self-stigma makes people adopt maladaptive coping strategies, such as avoiding stigmatizing situations (Hayward, Vartanian, and Pinkus 2018). This includes avoiding exercising with others (Hunger et al. 2015), which negatively impacts a person’s health. In combination with structural stigmatization, such as negative media portrayals, and interpersonal stigmatization, such as colleagues making remarks, this can lead to lower self-esteem and lower self-efficacy and consequently, people may become less motivated to achieve life goals in general (Corrigan, Larson, and Rüsch 2009). As a result, this reinforces norms and beliefs that stigma victims lack willpower or are lazy, thereby feeding motivations to stigmatize.

In this way, causes and effects of stigmatization can initiate a vicious circle on one level, triggering new vicious circles on other levels. In the reviewed literature on weight stigma we found vicious circles as a result of failure and success stories of treatment (Bacon and Aphramor 2011), stereotype anxiety and social consensus (Pearl 2018), changes in social network and reduced social support (Brewis 2014), no in-group favouritism (Tomiyama et al. 2015), stress and depression (Tomiyama 2014), low socioeconomic position (Hulshof et al. 2003), and stigmas on other traits (Lewis and van Puymbroeck 2008). This multitude of stigma drivers not only increases the pervasiveness of a health-related stigma but also makes it very persistent and increasingly challenging to deal with for stigma victims.

In conclusion, health-related stigma is a psychosocial phenomenon from which chronically ill individuals can hardly escape, as illustrated in Figure 3. The stigma often manifests itself on so many different levels with different actors that it maintains itself. Inspired by advancements in speculative design (Dunne and Raby 2013) and design fiction (Bleecker 2009), we think that design can play a role in stopping health-related stigmas from perpetuating themselves. In the following section, we argue for an approach in how to achieve this.

## Towards designing destigmatizing storytelling environments

Effectively tackling health-related stigma is difficult, as it is often very subtle and difficult to grasp (Cook et al. 2014). For example, Lupton (2018) argues that referring to obesity as an epidemic in itself is not stigmatizing, yet it does render large bodies susceptible for medical labelling. Due to the subtle nature of stigma, public health interventions sometimes even perpetuate it, because they fail to acknowledge that biases are a manifestation of social inequity (Alberga et al. 2016) or because the personal blame argument prevails throughout campaigns and policy (Lewis et al. 2011; de Boer and Lemke 2021).

To bring stigma perpetuation to a halt we suggest to redesign the social environments in which stigmatic interaction takes place, rather than contending against individual stigmatizing narratives or narrators. We can directly or indirectly influence the storytelling in these environments by creating an atmosphere or learning environment that makes participants feel safe, non-judgemental, and open to other perspectives. Within such a safe space, interactive narrative mechanics such as role playing and decision-making can provoke participants to interact with each other in a destigmatizing way. Such mechanics may support them to collaboratively determine the course of unfolding stories, change stories that already exist, or add new stories.

More specifically, designers can influence stigmatic storytelling or stimulate stigma-reducing storytelling by designing 1) the properties of a storytelling environment (e.g. Vaes et al. 2012) and 2) the rules that guide the storytelling interaction in an environment (e.g. Vegt et al. 2016). The first design strategy aims to influence participants’ storytelling through framing, i.e. ‘to select aspects of a perceived reality and make them more salient’ (Entman 1993, 52). For example, stories about obesity in the media were often accompanied by an image of a person with a large body with their head cut off (Heuer, McClure, and Puhl 2011). Image banks now provide images of people with large bodies in active and participating situations to foster recognition in stories about obesity. The second design strategy aims to evoke and steer storytelling by designing constraints and perceived affordances (Norman 1999). For example, predefined story elements and a stage and audience may guide participants towards desired or relevant storytelling.

### Narrative freedom as a persuasive dimension in storytelling environments

When creating or shaping the storytelling environment to reduce health-related stigma, ethical questions appear in relation to the narrative agency of participants (Meretoja 2017): which stories should dominate and who should get a voice in discourses where stigmas prevail? The answers to these questions vary over place and time. Hence, the participants in a storytelling environment end up continuously negotiating between values (Korthals Altes 2014).

A storytelling environment influences this value negotiation by setting the narrative freedom that participants have, i.e. to tell their stories in the way they want constraint by all possible stories that the environment allows. For example, a frame narrative that explicitly introduces the role of stigma perpetrator and stigma victim stimulates participants to adopt these two perspectives and interact accordingly. Moreover, narrative freedom arises from resources in the environment to tell stories and to be heard (Zussman 2012). For example, the audience that encourages someone to tell one’s story compromises narrative freedom through their consent, attention, agreement, involvement, and expertise (Pasupathi and Billitteri 2015). In a Black Lives Matter rally, for example, protesters tell other stories than when they sit around the dinner table with their families.

Another strategy of setting narrative freedom is to confine the degrees of freedom participants have in interacting in a storytelling environment. The field of interactive digital narrative has explored various ways to interact within a narrative (e.g. Koenitz 2023), thereby varying the distribution of narrative agency between a narrator and its audience. We can apply such interactive narrative design heuristics on the broader storytelling environment level. Such interventions would than lead to interactive storytelling environments in which participants have a designated freedom to create, present, reflect on, and feel part of stories. Participants may, for example, build on each other’s stories or adapt their own story after hearing someone else’s story.

In relation to stigmatization, balancing this narrative freedom is crucial from an ethical as well as utilitarian point of view. Storytellers need to have a certain degree of narrative freedom to become engaged in telling their story, yet within boundaries that foster stigma reduction. Too low narrative freedom might not trigger enough interaction between stigma actors. Too high narrative freedom might only amplify the status-quo in stigma manifestations, motivations, and health effects.

## Reducing health-related stigmas through storytelling

**Figure 4.**
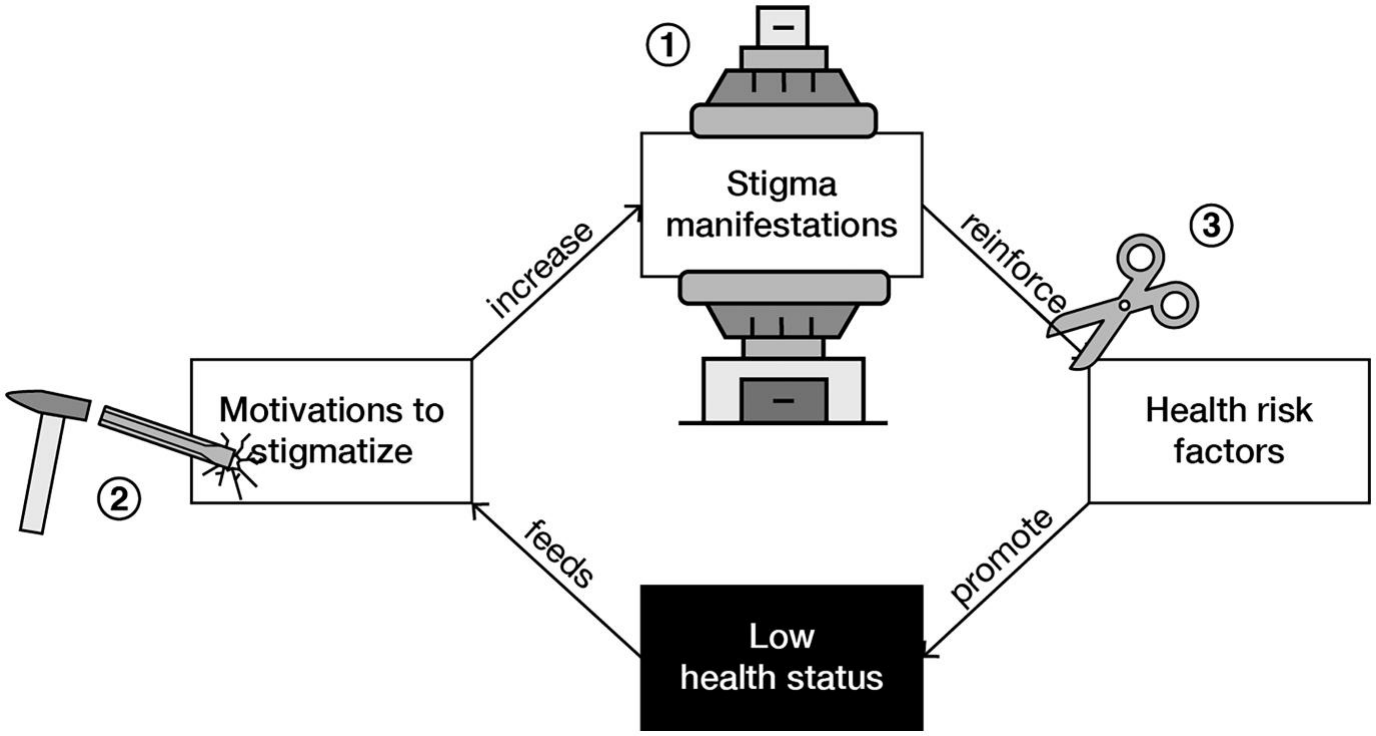
Three mechanisms to break the vicious circle in the health-related stigma perpetuation model. 1) Diminish stigma manifestations (press machine). 2) Deconstruct motivations (chisel and hammer). 3) deal with stigmatization (scissors).

Based on our model of stigma perpetuation (Figure 2), we identify three mechanisms that can break the vicious circle (as depicted in Figure 4). The first two mechanisms are to diminish stigma manifestations (mechanism 1) and deconstruct motivations to stigmatize (mechanism 2). This is supported by the claim of Link and Phelan (2001) that stigma can only be effectively reduced when the power of stigmatizing groups gets limited and when fundamental motivations to stigmatize (i.e. domination, norm enforcement, disease avoidance) are taken away. Richman and Lattanner (2014) add that stigma victims become empowered by promoting personal agency and control over the stigma. This supports the third mechanism of supporting stigma victims in dealing with stigmatizing situations to stop the reinforcement of health risk factors.

These stigma-reducing mechanisms may be well understood from a narrative perspective, as health-related stigma can be characterized as a complicated network of narratives that interact with each other and that perpetuate the stigma, as visualized in Figure 3. Storytelling may provide a way to intervene in this stigmatic narrative network by conveying new experiences, persuading the stigma actors, and exploring alternative perspectives (Meretoja 2017). To come to a more structured understanding of the potential destigmatizing functions of storytelling, we make a distinction between story content and story interaction. In the following sections, we further detail the potential functions of story content and story interactions in relation to the three mechanisms that could stop stigma perpetuation (see Table 1). In this way, we aim to describe a detailed research agenda regarding the destigmatizing potential of interactive storytelling environments.

**Table 1.** Seven potential functions of story content and story interactions in three basic stigma-reducing mechanisms.

Stigma-reducing mechanisms	Potential functions of story content	Potential functions of story interactions
Diminish manifestations	1) Expose victims and perpetrators to other perspectives	2) Provide a protective frame and 3) intervene in daily conversations
Deconstruct motivations	4) Reach stigma actors with persuasive messages	5) Exchange alternative understandings
Deal with stigmatization	6) Elicit understanding and support for stigma victims	7) Support stigma coping by stigma victims

### Diminish stigma manifestations

Interventions that aim to directly reduce health-related stigmatization, such as reducing bullying at schools, are often based on inducing empathy (Knaak, Modgill, and Patten 2014). For example, by explaining the difficulty of losing weight through first-person narratives (Teachman et al. 2003) and role-playing exercises (Wiese et al. 1992).

*1. Expose to other perspectives* – A basic precondition of reducing stigma through empathy is the availability of stories from different perspectives. Carefully distributing and defining these perspectives in a storytelling environment is important. With a clear presentation of scenes through the selection or restriction of narrative information (Gérard 1980), stories can be used to make stigma perpetrators experience the perspective of stigma victims and vice versa. Furthermore, the story medium should be chosen carefully to reach an audience with other perspectives. Davidson et al. (2018), for example, successfully reached the general public by publishing online video testimonies about mental illness among veterans and firefighters. When designing an interactive storytelling environment, it is thus advisable to already collect stories from varying perspectives and use those as prompts for destigmatizing discussions. Role playing and role shifting could be additional interactive narrative mechanisms that support participants in experiencing other perspectives.

*2. Provide a protective frame* – A protective frame of fiction is assumed to evoke empathic responses (Keen 2006; Kidd and Castano 2013). No conclusive evidence exists regarding the empathy-inducing effect of fictionality (Braddock and Dillard 2016). Yet, Carey et al. (2020) show that using a shared fiction does relieve participants to disclose very personal experiences and establish shared understandings of stigmatizing situations.

This effect is similar to what happens in gaming experiences. As described by Visch et al. (2013), gaming temporarily moves people’s attention away from their daily life. This mental transportation can lead to a psychological protective frame in which participants become more playful (Apter 1993) and feel stronger engagement, immersion, and emotional presence (Roth, Vorderer, and Klimmt 2009). If an interactive storytelling environment affords confidentiality or feels like a game in which exploration is allowed, stigma perpetrators may adopt a more open attitude towards their own stigmatizing behaviours. Additionally, such a protective frame may evoke a safer feeling among stigma victims to disclose personal stigmatic experiences.

*3. Intervene in daily conversations* – In daily conversations, stigma expressions could reduce by becoming aware of the underlying master narratives. Master narratives are ‘*culturally shared stories that guide thoughts, beliefs, values, and behaviours*’ (McLean and Syed 2015). This awareness may trigger or teach participants to counter stigmatic master narratives (Bamberg and Andrews 2004). An example of triggering counter narratives is the reframing of texts, such as ‘living with excess weight is not a sin, it is a quest’ (Vyncke and Van Gorp 2020). Participants can be triggered to counter stigmatic stories directly by providing them with ‘narrative ammunition’. For example, the labels imposed upon stigma victims, such as *healthy* and *sick*, almost never reflect reality. Being aware of such binary oppositions and normative choice of words and critiquing them (Derrida 1972) may support counter narratives in daily conversations. An interactive storytelling environment could provide triggers to reflect during conversations. Such ‘small story’ interventions not only affect the direct conversation but also enhance people’s reflexivity in general (Georgakopoulou 2015).

Another method that may fuel subversiveness towards stigmas is to recognize what is not being said (cf. Barthes and Duisit 1975). Health-related stigma is often felt the most when implicitly communicated, thus being able to recognize implicit messages in conversations may help in countering stigmatic narratives. Irigaray (1985) provides an example of deploying stories as *enacted*, as opposed to stories being *told*. For example, if women are viewed as illogical, they should speak logically about this view, thereby *enacting* the counter-narrative. Although critiqued for its relatively aggressive approach (Kozel 1996), an interactive storytelling environment could support stigma victims in enacting counter stories through, for example, theatrical props.

### Deconstruct motivations to stigmatize

The mechanism of deconstructing stigma motivations entails changes in stigmatic beliefs, attitudes, and intentions, such as the idea that an illness is fully controllable by the individual that is ill. Motivations behind obesity stigma, for example, may be altered through stories about multiple factors, not only lifestyle, that lead to weight gain. Destigmatizing education of healthcare practitioners is mainly aimed at altering motivations (Knaak, Modgill, and Patten 2014), such as stories about the noncontrollable causes of an illness (e.g. Puhl, Schwartz, and Brownell 2005) and the causes and effects of health-related stigma itself (e.g. Hague and White 2005).

*4. Persuade stigma actors –* The persuasive strength of such stories is key to alter motivations to stigmatize (De Graaf, Sanders, and Hoeken 2016). Van Laer et al. (2013) describe that the persuasiveness of stories depends on narrative transportation, i.e. experiencing story events through feeling the emotions of a story character and vivid images of the story plot. Hence, a persuasive storytelling environment should enable participants to identify with story characters (e.g. Winskell, Sabben, and Obong’o 2019) and easily imaginable events, such as anecdotes and daily life situations (Boeijinga, Hoeken, and Sanders 2017) in familiar cultural contexts (Larkey and Hecht 2010). Another characteristic of narrative persuasion is that it predominantly has story-consistent effects (Braddock and Dillard 2016). Thus embedding destigmatizing messages and showing destigmatizing behaviour in a story is more likely to reduce stigma than showing stigmatic behaviour or literally stating the message of a story (Shen, Sheer, and Li 2015).

*5. Exchange alternative understandings* – Next to persuasive stories, we suggest feeding storytelling environments with alternative understandings. This proposal builds on social consensus mechanisms that have been studied as a destigmatizing intervention. Puhl, Schwartz, and Brownell (2005) demonstrated that stigmatic attitudes of undergraduate students diminished when being exposed to positive attitudes of peers. Another strategy to evoke alternative views in storytelling environments is to value disagreement and pluralism, i.e. to adopt an agnostic approach (Mouffe 1999). Accordingly, variation in stories should be the denominator for distributing storytelling resources, rather than supporting every storyteller equally. Moreover, the content and message of a story should not be judged but responded to with other stories. As a result, participants in storytelling environments ideally alternate between storyteller and listener roles (cf. Hammond, Pain, and Smith 2007). Still, stigmatic stories may be adopted as easily as destigmatizing stories (Cavazza et al. 2015). Hence, in addition to agnostic resource distribution, storytelling environments should actively promote destigmatizing storytelling or penalize stigmatic stories.

### Deal with stigmatization

The mechanism of dealing with stigmatization builds on literature describing how, predominantly female, stigma victims themselves develop their ways of dealing or coping with health-related stigma. First of all, ways to deal with self-stigma come down to maintaining or fostering self-esteem and self-worth, such as depersonalizing the stigma (Bombak and Monaghan 2017), finding social support (Chou, Prestin, and Kunath 2014), focus on body functionality (Alleva et al. 2015), and positive self-talk, self-love, and self-acceptance (Myers and Rosen 1999). Other examples of coping strategies for stigma victims are fighting back, rationalizing the stigmatizing behaviour as a flaw from the perpetrator, feeling competent regarding the illness, refusing to hide the illness and ignoring the stigmatizing situation (Myers and Rosen 1999; Lewis et al. 2011; van Amsterdam and van Eck 2019; Lu et al. 2003). These activities can all be supported with storytelling.

*6. Elicit understanding and support* - Ziebland and Wyke (2012) suggest that stigma mainly reduces by learning from experiences from others. Moreover, tailoring stories to stigma victims and adopting diversity values are assumed to have an empowering effect (Reid et al. 2014). At the same time stories should address the perspective of stigma perpetrators to exert attitudinal and behavioural change (Larkey and Hecht 2010). Consequently, there is a duality between empowering stigma victims and persuading stigma perpetrators.

Explicating the roles of victim and perpetrator in a storytelling environment may solve this duality. Bruneau and Saxe (2012) show that a positive attitude change happens when members of a low power group tell their perspective to members of a powerful group. Additionally, powerful group members show positive attitude changes towards the low power group after summarizing what they have heard. Hence, explicitly assigning stigma victims as storyteller and perpetrators as listener elicits understanding and support for both sides. Consequently, learning to tell the story helps to deal with stigmatization. Being able to align one’s story with other narratives and thus telling one’s experiences appropriately helps to elicit understanding and support from others.

*7. Support stigma coping* – The process of constructing a coherent story may support stigma coping as well. Storytelling with others can help in making sense of what has happened, thereby supporting the reflective process of dealing with stigmatizing situations (Lely et al. 2019). On an intrapersonal level, co-created stories may benefit education, counselling, expressive writing, belonging, and values affirmation (Cook et al. 2014). On an interpersonal level, collaborative storytelling could enhance coping in therapy group sessions, patient-physician interactions, parent-child dialogue, and peer support groups (Winskell, Sabben, and Obong’o 2019). Collaborative storytelling could support stigma coping on a structural level by initiating policy and design changes, and by providing role models (Gomillion and Giuliano 2011).

## Demonstration of an interactive storytelling environment that reduces weight stigma

**Figure 5.**
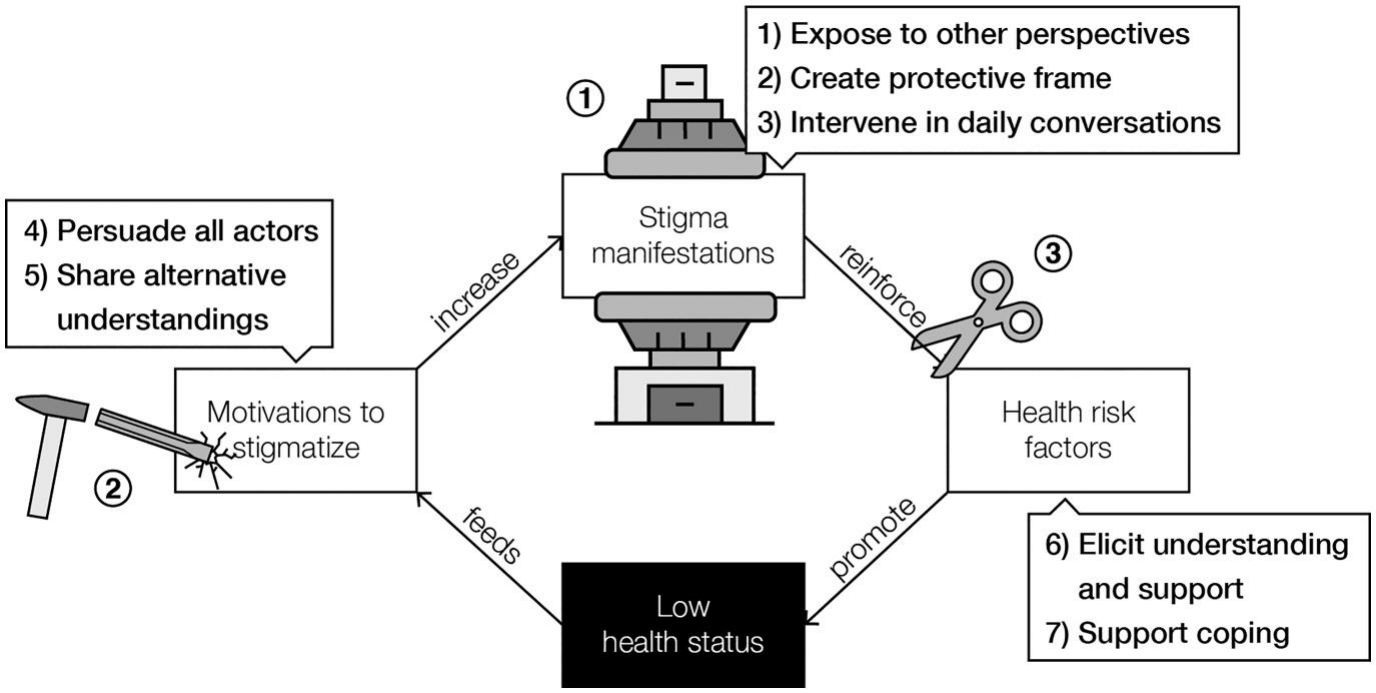
Seven functions of interactive storytelling environments intervening on three mechanisms in the self-perpetuation of stigma.

**Figure 6.**
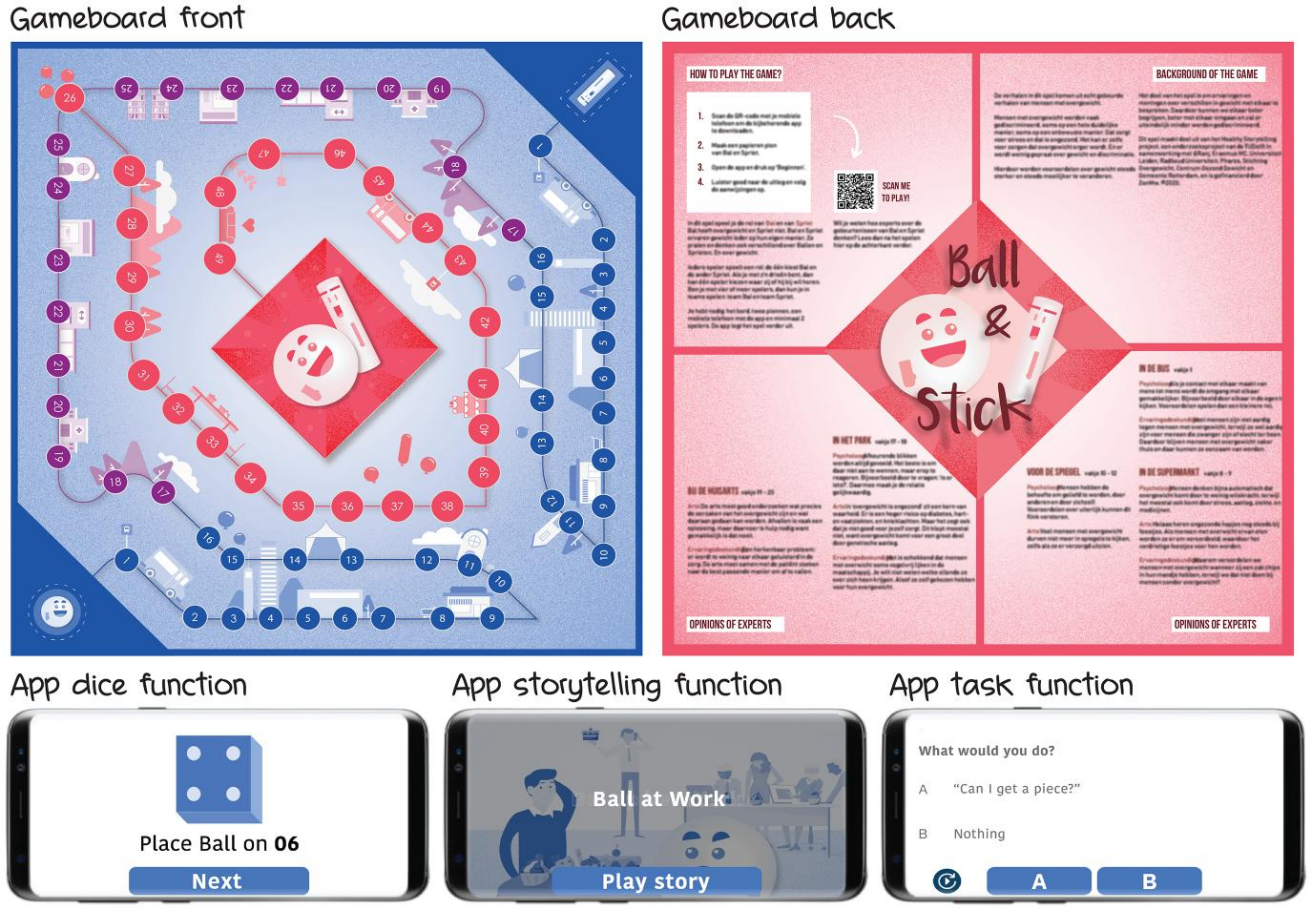
The game of Ball & Stick: a physical board game with mobile application.

To break the vicious circles of health-related stigma, interactive storytelling environments ideally deploy all above-described destigmatizing functions of storytelling. As shown in Figure 5, storytelling environments should 1) expose stigma victims and perpetrators to other perspectives, 2) provide a protective frame, 3) intervene in daily conversations, 4) persuade all stigma actors, 5) exchange alternative understandings, 6) elicit understanding and support for stigma victims, and 7) support stigma victims to cope with stigmatization. In this section we give an example of an interactive storytelling environment against obesity stigma: the game of Ball & Stick. It is not meant as a full case study, but to demonstrate how the above-described theory could be applied (Figure 6).

### Ball & Stick

Ball & Stick is a game for community centres with the purpose of reducing the stigmatization of people with obesity. Ideally, it is played with four participants. The game consists of a gameboard, two pawns, and a mobile application that verbally guides players through the game with a virtual dice, narrated stories, and discussion tasks (see Appendix for a detailed content description). The story in the game revolves around two abstractly visualized characters (function 2) represented by two pawns: Ball – a stigma victim, and Stick – a stigma perpetrator. Participants choose which role they want to play. During the game, the players are confronted with stigmatizing situations that people with obesity encounter (function 1). The game facilitates a careful build-up of the discussion between the players through increasing narrative freedom by first choosing between response options, then generating a personal response, and finally jointly agreeing on a response. This varying narrative freedom guides the players in stepwise formulating their thoughts (function 5).

All in-game events are based on conversations with community centre visitors, people with obesity, and obesity specialists. In this way, the events in the game are familiar and easily imaginable (function 4). Additionally, narrative transportation is eased through the common overall narrative of a love story and the game rules were as simple as game of goose. The in-game stigmatic situations were based on regular daily life events, including commonly occurring conversations (function 3). The collected real stories are all from the stigma victim perspective so that the game basically serves as a message from stigma victims to stigma perpetrators (function 6). To allow for coping, next to raising awareness, the back of the gameboard features expert interpretations of several events in the game (function 7). Moreover, the closing narration in the app explicitly states the coping strategy of talking to each other about obesity stigma.

We evaluated the Ball & Stick game with 22 visitors of two community centres (age: 60 (43-83), female: 15) in low-income neighbourhoods of two Dutch cities. We targeted the game to middle aged people with a low income because low socio-economic position is significantly related to obesity prevalence and obesity often develops when people finish their working life (Robroek et al. 2015). Five groups of four players and one group of two played the game in a meeting room at the two community centres. Fourteen of the twenty-two participants lived in neighbourhoods that are within the 10% poorest neighbourhoods in the Netherlands and approximately half of the group had overweight (based on observation).

After a short introduction and giving informed consent the game session started. The two present researchers gave no instructions, as the game was designed to be standalone. Researchers would only intervene in case of a technical malfunction and occasionally encouraged participants to speak more clearly. After playing the game, the players started a discussion, sometimes directly triggered by the game. After their first reactions, the participants were asked to fill in a questionnaire and the researchers initiated a group discussion about the usability, experience, and effect of the game.

Nearly all participants reported a positive engaging experience. Many indicated that the events in the game were familiar to them and that the game raised their awareness. For sixteen participants the game made it easier to talk about obesity and twelve said that it changed their attitude towards people with obesity.

A significant effect of the game was the self-initiated discussion among participants after gameplay. Several groups discussed causes and solutions for discrimination, obesity, and healthy eating. Personal stories about racism, burnout, and depression, suggest that the game created an open atmosphere between the players in which varying beliefs and perspectives in relation to obesity stigma were exchanged. Many participants recognized events from their own experience and explicitly shared their personal beliefs, that were sometimes challenged by others. Moreover, participants explained that the game taught them the basic mechanisms of stigmatization. Yet they also indicated that these positive conversations probably only happen during the game and that they will ‘call each other “fatty” again’ as soon as they leave the room. Nonetheless, we conclude that the Ball & Stick game was successful in creating a storytelling environment for destigmatizing interaction between people with varying experiences, perspectives, and beliefs.

## Discussion

In this article we introduce the concept of *interactive storytelling environments* as a means to reduce health-related stigmatization and suggest applying it to places where health-related stigma is expressed, such as bullying at community centres, judgmental recommendations at supermarkets, or blunt instructions at hospitals. Within interactive storytelling environments, participants are invited to listen and respond to stories from others and at the same time guided in telling their own. As demonstrated by the Ball & Stick game, the advantage of interactive storytelling environments is that they can be deliberately designed to evoke empathy with others, stimulate discussion and critical thinking, and guide towards consensus.

This approach could be a valuable addition to current speculative design approaches in which products and objects are designed to encourage critical reflection (Dunne and Raby 2013). Provocative storytelling environments can stimulate critical reflection and facilitate interactions between different stigma actors. The narrative freedom for each actor is a major parameter in such environments to influence the storytelling possibilities that stigma actors have. By designing interventions that afford or limit this freedom in storytelling, stigma actors can be directed towards 1) diminishing stigma manifestations, 2) deconstructing motivations to stigmatize, and 3) dealing with stigmatizing situations.

The benefit of interactive storytelling environments is not only restricted to health-related stigma. It can also be used to tackle other types of stigmas. Many social issues, such as women’s rights, attitudes towards migrants, and racism could be addressed with design interventions using the storytelling environment approach. More generally, many design processes could benefit from a storytelling environment perspective, in particular where vulnerable or minority groups are involved (Parrott, Carpentier, and Northup 2017). On the one hand storytelling feeds the design process (e.g. Maxwell et al. 2015) and on the other hand a deliberately designed environment can guide the storytelling.

Within the limitations of this paper, we could not give an exhaustive overview of storytelling theories, yet with the seven functions of a storytelling environment we aim to set a research agenda regarding its destigmatizing potential. The Ball & Stick case provides a first idea of designing and applying interactive storytelling environments and demonstrates that such environments contribute to health-related stigma reduction. To apply it more broadly a generic model and design method are required. The basic narratological and interactive digital narrative concepts that we briefly touched upon in this article could serve as a basis for such a model, which could also take further the field of applied narratology (Moenandar 2018). Moreover, knowledge from participatory design and user generated content (cf. Lukyanenko et al. 2016) could support the practical application of such a model.

The seven stigma-reducing functions of storytelling may be applied to a large variation of health-related storytelling environments, for example in the conversation between patients and physicians in consultation rooms. In such contexts, a narrative protective frame could comfort patients and support physicians. Moreover, to welcome storytelling in such a situation could evoke both parties’ openness to the other’s perspective and enable exchange of alternative understandings (Bury 2001). Storytelling tools, such as props or preformatted texts, could help to deliberate on the use of words, as well as deploy the persuasive qualities of narrative transportation. And to make it complete, the consultation room should provide patients with the tools to tell their story appropriately to elicit understanding from the physician and to counter possible unfortunate events of stigmatizing behaviour.

Eventually, the key role of an interactive storytelling environment is to activate a conversation between all stigma actors and guide them towards destigmatizing storytelling. To achieve this, questions about the properties of an interactive storytelling environment pop up. In this article, we covered narrative freedom as a core dimension. Other important properties would include the composition and interests of participants, modes of communication (e.g. verbal, visual, theatrical), and the literacy level of participants. To connect with the participants’ interests and experiences we envision a co-creative process in which all users of a storytelling environment are involved in creating the stories. For example, Whitley et al. (2020) show that documentary-style videos about mental illness, created by people with a mental illness, led to informative, relatable, attention-grabbing, and change-inducing videos that have promising stigma-reducing effects among the viewers.

Finally, we think that interactive storytelling environments contribute to the transition in healthcare from paternalistic top-down approaches to deliberative patient-centred bottom-up approaches (Emanuel and Emanuel 1992). Accordingly, designers of storytelling environments should maintain a neutral position and focus on providing a fitting storytelling environment that stimulates empathy and an awareness and exchange of values and ideas. The environment should mainly facilitate a fair process of meaning-making among all health-related stigma actors, trusting that nearly everyone can tell stories and listen to them.

## Conclusion

In this article we propose to use interactive storytelling environments to reduce health-related stigma. We first developed an understanding of the mechanisms behind stigmatization with the health-related stigma perpetuation model (Figure 2) to come to mechanisms on how to break the vicious circle. Due to the multi-layered and multifaceted character of stigmas, we suggest influencing stigmatic story*telling*. We theorize seven stigma-reducing functions of storytelling that can be applied to social environments and discuss design considerations within these functions. Some of these considerations have been applied to a design case that demonstrates a destigmatizing interactive storytelling environment: the game of Ball & Stick.

In conclusion, this paper is mainly a call to designers, researchers, and practitioners in health and wellbeing to become aware of health-related stigmatization and create the conditions to reduce stigmas. Our hope is that our propositions in this paper inspire to further erase stigmas by design.

## Appendix:

### Content of the game of ball & stick

**Table:**

Introduction story
This is the story of Ball and Stick. It is a fantasy story, but the events come from true stories that people experienced in real life. Ball has overweight and Stick does not. Ball and Stick experience their weight in their own way. They also talk and think differently about balls and sticks. And about weight. People with overweight are regularly discriminated against. This causes stress. And stress is unhealthy. It increases the problem of being overweight. The goal of this game is to increase your knowledge about discrimination on weight. And how you can reduce it. In this game, Ball and Stick experience many different situations. You have to answer a question in each situation. There are no right or wrong answers and there will be no winner. The game finishes when you’ve reached the end together. But how do you do this? You play the game with two players. Both of you have a role; one can choose Ball and the other Stick. If you’re with three, then one player can choose which role to join. If you’re with four players or more, you can play in teams: team Ball and team Stick. Make a paper pawn of Ball and Stick. Place Ball and Stick on position one. Ball begins. Press “Start”.
Dilemmas
Ball steps onto a crowded bus. There is only one narrow seat left. What do you do? Take a seat and be sandwiched with other passengers. Keep standing with pain in your back.
Stick steps onto a full bus. There is only one seat left next to someone who takes a lot of space. What do you do? Sit against him or her. Keep standing.
Ball is standing in front of an elevator and hears others thinking: “such balls should take the stairs”. What do you do? Don’t care about what others are thinking Explain that you have a knee injury.
Stick is stepping into an elevator. Stick sees someone approaching who is clearly overweight. What do you think? “It would be better if that person would take the stairs.” “There’s someone coming, I’ll keep the door open.”
Ball is at work. Someone is serving cake. Ball is being skipped and doesn’t like this. What do you do? Ask for a small piece Nothing, because you feel ashamed.
Stick is at work. It’s Stick’s birthday, so Stick is serving cake. What do you do? Not tempt the colleague with overweight and serve an alternative treat. Ask if the colleague with overweight would like a piece of cake.
Ball is at the supermarket. Chips and soda are on discount, so Ball buys chips and soda. In the cue at the cash desk someone looks disapprovingly at the groceries. What do you say? “This is not for me.” “Mind your own business”.
Stick walks through the supermarket. Stick sees a person that is overweight buying chips and soda. What do you think? “That person buys very unhealthy stuff!” “Nice! That person is throwing a party.”
Ball is alone at home and looks in the mirror. What do you think? “I need to put on beautiful clothes, so I will look thinner.” “I look good.”
Stick is alone at home and looks in the mirror. Stick has gained some weight. What do you think? “I will lose weight naturally, I don’t need to do anything about it.” “It’s time to go to the gym again, then I’ll lose those kilos in no time.”
Ball is at a party and sees Stick. What do you think? “Such a skinny and cheerless person.” “Nice to meet someone different.”
Stick is at a party and sees Ball standing next to the snacks. What do you think? “Nice party.” “Always the same people standing close to the snacks.”
Ball is at a party talking with some other balls. Ball sees Stick looking at Ball. What do you think? “Stick probably thinks I am ugly, I will not approach Stick.” “Stick likes me, I will go sit next to Stick.”
Stick is at a party sitting on the couch. Ball is attracting Stick’s attention. What do you think? “Ball is not like me. That’s not going to work out.” “Ball is different. I would like to get to know Ball.”
Open response task
Ball and Ball’s partner are walking through the park. They see Stick walking. “All those skinny smart asses, yet they look very unhealthy”, says Ball’s partner. *How would you react to this? Form your opinion and tell it in 20 seconds to the other.*
Stick and Stick’s partner are walking in the park. They see Ball walking. Stick’s partner is pointing at Ball. “Those balls always look very unhealthy.” *How would you react to this? Form your opinion and tell it in 20 seconds to the other.*
Ball is at the doctor for pain in the knee. The doctor says: “Go first lose some weight.” *How would you react to this? Form your opinion and tell it in 20 seconds to the other.*
Stick is at the doctor for a sore knee. The doctor says: “There’s nothing you can do about it. Here’s a prescription for painkillers”. *How would you react to this? Form your opinion and tell it in 20 seconds to the other.*
Ball and the partner of Ball are running on a treadmill at the gym. Ball is exhausted and stops. Ball’s partner says: “Just continue, lazy ass!”. *How would you react to this? Form your opinion and tell it in 20 seconds to the other.*
Stick and Stick’s partner are at the gym. They see Ball doing exercises. Stick’s partner says: “I bet that ball will drop out any minute now”. *How would you react to this? Form your opinion and tell it in 20 seconds to the other.*
Ball is standing next to a plus-size rack in a clothing shop. Ball thinks: “Why is the plus-size clothing on a rack separate from the other sizes?” *How would you react to this? Form your opinion and tell it in 20 seconds to the other.*
Stick is standing in a clothing store. Stick sees a rack of plus-size pants. Stick thinks: “Why can’t those balls just lose some weight?” *How would you react to this? Form your opinion and tell it in 20 seconds to the other.*
The partner of Ball is coming home from work. Ball’s partner says: “Finding a job is a lot easier for Sticks than for us.” *How would you react to this? Form your opinion and tell it in 20 seconds to the other.*
Stick and Stick’s partner are at a library. Stick’s partner says: “Balls are stupid, they think that paprika crisps are healthy.” *How would you react to this? Form your opinion and tell it in 20 seconds to the other.*
Ball is at a party. Ball sees Stick sitting on the couch. Ball joins Stick and thinks: “With Stick I do dare to talk about my weight.”
Stick is at a party. Stick sees Ball sitting on the couch. Stick joins Ball and thinks: “With Ball I can have conversations that are not only about how I look.”
Making choices together
Ball and Stick want to do sports together in the park. *How do you do this? Choose a way of doing sports without saying it to the other:* Stick runs too fast, so they go separate from each other. They walk slowly, so Ball can keep up. Stick goes running and Ball cycles along. *Did you both choose for the same option? If not, choose again for a shared answer.*
Ball and Stick prepared dinner together. They want to add a drink that tastes good and is healthy. *Which drink will you choose? Choose a drink without saying it to the other:* Water. Alcohol-free beer. Fresh orange juice. *Did you both choose for the same option? If not, choose again for a shared answer.*
Stick and Ball organize a party. With balls and sticks mingled together. One of the balls greets Ball: “Hi chubby! How are you?” *How would you call someone with overweight? Choose for a word without saying it to the other.* Fat. Someone with obesity. *Think of another word*. *Did you both choose for the same option? If not, choose again for a shared answer.*
Ball and Stick are invited for a wedding with a lot of food. They are not sure if they should go. *Why do you hesitate? Choose a reason without saying it to the other:* Because they are afraid to get nasty comments. Because they don’t want to eat too much. Because the seats might be too narrow. *Did you both choose for the same option? If not, choose again for a shared answer.*
Ball and Stick go on a trip by bus to Zeeland. One seat costs 10 euro. *What do you do? Choose what you would do without saying it to the other:* Book an extra seat. Sit tight. Stay home. *Did you both choose for the same option? If not, choose again for a shared answer.*
Ball and Stick are sitting on the couch at home. Ball says: “I love you Stick”. “I love you too”, says Stick. *How do you think about love? Choose a sentence without saying it to the other:* Love is cooking delicious meals for each other. Love is letting the other win in a game. *think of a sentence yourself.* *Did you both choose for the same option? If not, choose again for a shared answer.*
Ending story
Congratulations! You’ve reached the end! The story started with Ball and Stick stepping into a bus. Did you choose to sit? Or rather not? Ball and Stick sometimes did see each other, but they didn’t talk to each other. Then Ball and Stick meet each other at a party. They mainly notice each other’s differences. Among the other Balls and Sticks they encounter many false beliefs. They point at each other and judge each other. But Ball and Stick start to realize that the differences are not so large at all. Being fatter or slimmer is just the way it is. By talking to each other Ball and Stick understand each other better. They also understand other balls and sticks better now. Were you able to better understand Ball and Stick? Did you notice that you also judge people with overweight sometimes? Maybe without being conscious about it? And do people with overweight know that others often have good intentions? We hope that, after playing this game, you can now better deal with differences in weight without making others feel bad about it. Just like Ball and Stick!
